# Effects of Mechano-Electric Feedback on Scroll Wave Stability in Human Ventricular Fibrillation

**DOI:** 10.1371/journal.pone.0060287

**Published:** 2013-04-03

**Authors:** Yuxuan Hu, Viatcheslav Gurev, Jason Constantino, Jason D. Bayer, Natalia A. Trayanova

**Affiliations:** 1 Department of Biomedical Engineering, Johns Hopkins University, Baltimore, Maryland, United States of America; 2 Functional Genomics and Systems Biology, IBM T. J. Watson Research Center, Yorktown Heights, New York, United States of America; University of Minnesota, United States of America

## Abstract

Recruitment of stretch-activated channels, one of the mechanisms of mechano-electric feedback, has been shown to influence the stability of scroll waves, the waves that underlie reentrant arrhythmias. However, a comprehensive study to examine the effects of recruitment of stretch-activated channels with different reversal potentials and conductances on scroll wave stability has not been undertaken; the mechanisms by which stretch-activated channel opening alters scroll wave stability are also not well understood. The goals of this study were to test the hypothesis that recruitment of stretch-activated channels affects scroll wave stability differently depending on stretch-activated channel reversal potential and channel conductance, and to uncover the relevant mechanisms underlying the observed behaviors. We developed a strongly-coupled model of human ventricular electromechanics that incorporated human ventricular geometry and fiber and sheet orientation reconstructed from MR and diffusion tensor MR images. Since a wide variety of reversal potentials and channel conductances have been reported for stretch-activated channels, two reversal potentials, −60 mV and −10 mV, and a range of channel conductances (0 to 0.07 mS/µF) were implemented. Opening of stretch-activated channels with a reversal potential of −60 mV diminished scroll wave breakup for all values of conductances by flattening heterogeneously the action potential duration restitution curve. Opening of stretch-activated channels with a reversal potential of −10 mV inhibited partially scroll wave breakup at low conductance values (from 0.02 to 0.04 mS/µF) by flattening heterogeneously the conduction velocity restitution relation. For large conductance values (>0.05 mS/µF), recruitment of stretch-activated channels with a reversal potential of −10 mV did not reduce the likelihood of scroll wave breakup because Na channel inactivation in regions of large stretch led to conduction block, which counteracted the increased scroll wave stability due to an overall flatter conduction velocity restitution.

## Introduction

Experimental and clinical research has demonstrated that the mechanical environment of the heart, in health and disease, is capable of exerting influence on cardiac electrophysiology [1]. Temporal changes in strain take place during all phases of the cardiac cycle. Abnormal electrical propagation during arrhythmias also leads to abnormal strain distributions in the heart, which in turn could affect electrical propagation. The mechanisms that contribute to strain-dependent modulation of electrical wave propagation are termed mechano-electric feedback (MEF) mechanisms [2].

There are several MEF mechanisms in the heart, including stretch-induced changes in intracellular Ca handling [3], depolarization of cardiac fibroblasts by stretch (via mechano-sensitive ion channels) affecting the resting potential and action potential duration (APD) of the coupled myocyte [4], and most importantly, myocyte sarcolemmal channel activation by mechanical stimuli [5], [6]. Stretch-activated channels (SAC), a type of mechanically activated ionic channels identified in cardiac tissue, have been found responsible for the generation of arrhythmias following an appropriately timed mechanical impact to the heart (commotio cordis) [7], [8], as well for the termination of ventricular arrhythmias following a precordial thump [9]. Abnormal deformation associated with the establishment of arrhythmia can also affect the progression of the arrhythmia itself; this aspect of MEF has received less attention in the literature.

On one hand, opening of SAC has been demonstrated to depolarize the resting membrane and thus cause Na channel inactivation [10], [11], which can stabilize scroll waves [12], the waves that underlie reentrant arrhythmias. On the other hand, SAC-induced depolarization and Na channel inactivation have been shown to give rise to scroll wave breakup [13] that increases electrical instability and leads to turbulent behavior underling the most lethal arrhythmias. These contradictory results indicate that the conditions under which and the mechanisms by which recruitment of SAC alters scroll wave stability remain incompletely understood.

To provide a comprehensive understanding of the mechanisms by which SAC opening affects the stability of scroll waves, it is necessary to record, in 3D, both the electrical and mechanical activity simultaneously, and at a high spatiotemporal resolution. Currently, this is not possible by means of experimentation. In contrast, biophysically-detailed computer simulations of electromechanical function at the organ scale have the capability to dissect the relationship between stretch and arrhythmia maintenance; initial attempts in this direction have already been made [14], [15]. The latter simulation studies had focused predominantly on the effect of recruitment of SACs with large conductances and reversal potentials close to zero on scroll wave stability. However, SACs have been demonstrated to exhibit a wide variety of reversal potentials and conductances [5]. Opening of SAC with a reversal potential close to the resting membrane potential has been shown to shorten APD [16], while opening of SAC with a less negative reversal potential has been found to have the opposite effect, resulting in APD prolongation [17]. The degree of lengthening or shortening of APD is affected by SAC conductance as well. Since recruitment of SAC with different reversal potentials and conductances leads to different electrophysiological changes in cardiac myocytes, it is thus possible that SAC opening could affect scroll wave stability in the 3D heart via different mechanisms depending on the channel population characteristics.

The goal of this study was to conduct a comprehensive analysis of the effects of SAC recruitment on scroll wave stability in the fibrillating ventricles. To achieve this goal, a strongly-coupled MRI-based biophysically-detailed electromechanics model of the human ventricles was developed. We used this model (1) to test the hypothesis that recruitment of SAC affects scroll wave stability differently depending on the reversal potential and channel conductance of SAC and (2) to uncover the relevant mechanisms underlying the different behaviors.

## Materials and Methods

### Electromechanical Model

**Figure 1:**
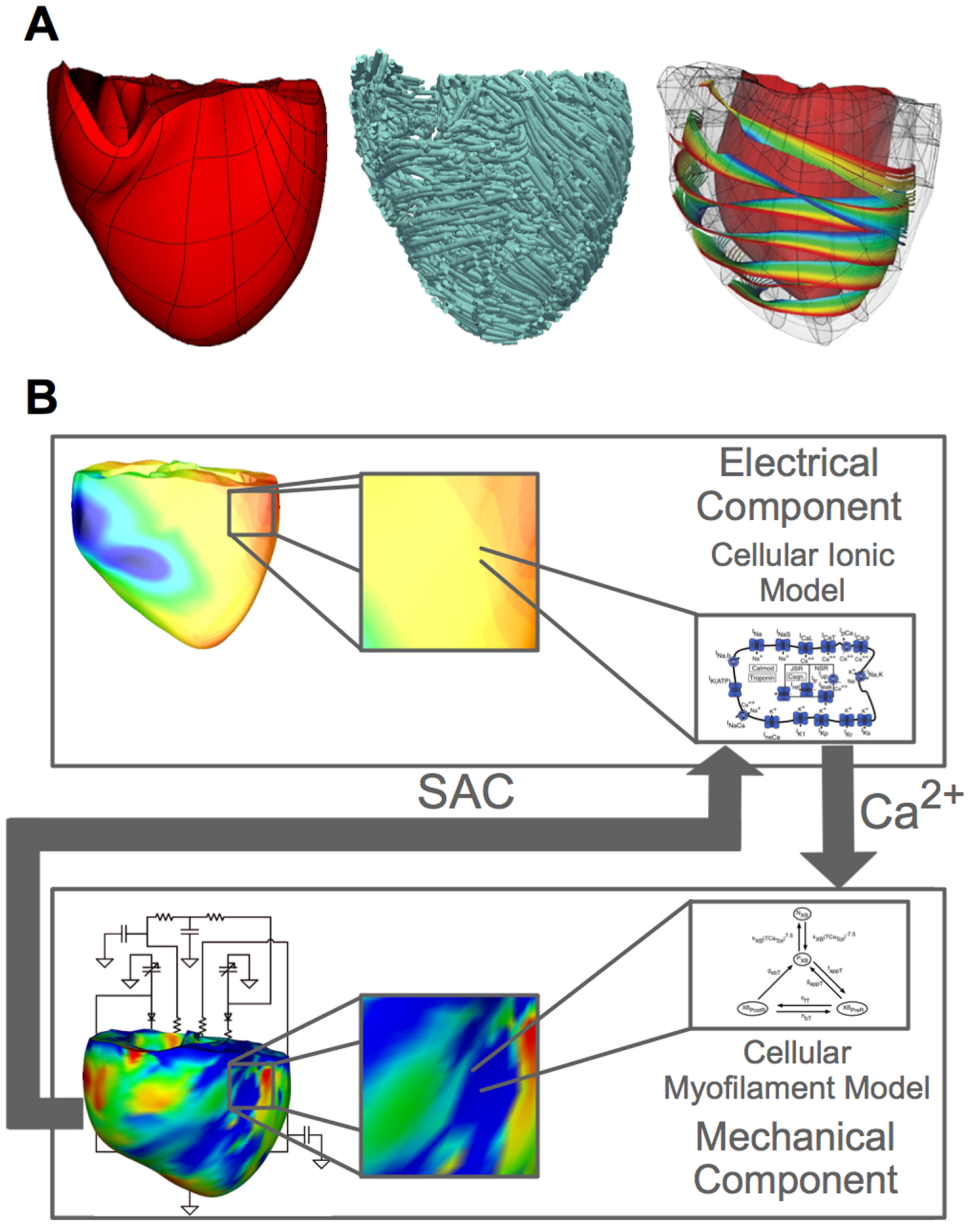
MRI-based electromechanical model of the human ventricles. (A): The mechanical mesh, fiber orientation and sheet structure of the human ventricular model. (B): The schematic diagram of the electromechanical model.

The image-based 3D electromechanical model of the human ventricles developed for this study as shown in Figure 1A incorporates realistic ventricular geometry and fiber-sheet architecture reconstructed from high-resolution magnetic resonance (MR) and diffusion tensor MR images [18]. The model consists of an electrical and a mechanical component (Figure 1B), which are coupled via the intracellular calcium dynamics [19], [20].

Mathematical description of the electrical component of the model was based on the monodomain representation of cardiac tissue; our group has made extensive use of such large-scale electrophysiological models of the heart [21]. Membrane kinetics were represented by the ten Tusscher et al. ionic model of the human ventricular myocyte [22]. To make the substrate prone to arrhythmia, the maximum conductances of the IKr, IKs, IpCa, and IpK currents and the time constant for the f gate of the L-type calcium current were modified throughout the ventricles, as described in [23], producing a steep local APD restitution curve with a maximum slope of 1.8.

The mechanical component incorporated a continuum mechanics model of the ventricles and a lumped-parameter model of the circulatory system, both of which have been described previously [19]. The parameters in the circulatory system model were adjusted for the human ventricles using available physiological data [24]; parameter values are presented in the Supporting Information Table S1. Active tension generation in the mechanics component was represented by the Rice et al. model of myofilament dynamics [25]. To simulate reduced contractility during arrhythmias, the half-activation constant for shift of a regulatory unit to a permissive state in the Rice et al. model was increased by 20% to decrease the sensitivity of troponin to Ca; this ensured that the maximal pressure during ventricular fibrillation (VF) matched that observed clinically [26].

The electrical mesh consisted of 4274379 elements with a spatial resolution of 500 µm; our electrical meshes are always tested for convergence (for the specific solvers we used; descriptions can be found in [27]). Description of the electrical mesh generation procedure can be found in Prassl et al. [28] The mechanical mesh [19] was a nonlinear mesh with 230 hexahedral elements. The spatial resolution for the mechanical mesh was 10 mm. The methodology for the generation of the mechanical mesh is described in Gurev et al [19].

The electrical and mechanical components of the model were strongly coupled. A mechanical solution step (500 µs) followed every five electrical solution steps (100 µs). During electrical propagation, the spatial distribution of intracellular Ca concentration throughout the ventricles was calculated from the ionic model at each node in the electrical component computational mesh and then, at every fifth solution step, mapped onto the Gaussian points in the mechanical computational mesh. At every Gaussian point, the local intracellular Ca concentration was inputted into the myofilament model to generate the local active tension. After solving for the mechanical deformation of the ventricles arising from the active tension, strain tensors were mapped back onto the nodes in the electrical computational mesh. From the strain tensor at each node, the local stretch ratio in the fiber direction was determined and used to calculate the local SAC current (see below for formulation) at that node, which in turn affected global propagation. The electromechanical model detailed above has been extensively validated by our group using electromechanical wave imaging [29]. Numerical approaches to solving the equations of the electromechanical model used methodologies described elsewhere [19], [30]. This state-of-the-art human electromechanical model is of broad applicability and can be used in a wide range of studies in cardiac electromechanics.

### MEF

Consistent with the goal of the study, SACs were incorporated in the electromechanical model of the human ventricles; opening of SAC was the mechanism by which mechanical deformation provided feedback into the electrical activity of the heart. The possible effect of mechanical deformation on geometry was not incorporated into the model to decrease model complexity and because it has been demonstrated to not affect scroll wave stability [15]. Since the current through SAC, I_SAC_, has been shown to increase linearly with stretch [17], I_SAC_ was formulated as being linearly proportional to the stretch ratio in the fiber direction, λ_f_:
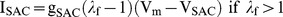


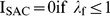
(1)where V_m_ is the transmembrane potential, and V_SAC_ is the reversal potential of SAC. SACs were assumed to conduct only when λ_f_ was larger than 1 [31]; thus, I_SAC_ was zero during myofiber shortening. Since I_SAC_ is the total current through both non-selective cation and potassium-conducting SACs, the value of V_SAC_ depends on the degree of the relative expressions of non-selective cation and potassium-conducting channels in myocardial tissue. Given that non-selective cation SACs have been reported to have a reversal potential of 0 mV [32] and potassium-conducting SACs operate with a reversal potential of −90 mV [33], V_SAC_ can range between −90 mV and 0 mV. In this study, we used two values of V_SAC_ that spanned that range, one close to the membrane resting potential (−60 mV [34]) and another less negative (−10 mV [17]). The SAC conductance g_SAC_ was varied between 0 and 0.07 mS/µF [1], [35], [36] to fully investigate the effects of SAC recruitment on scroll wave stability.

### VF Induction Protocol

To induce VF, the ventricles were first paced seven times from the apex at a 700 ms basic cycle length to achieve steady-state propagation. Then, at 500 ms following the last pacing beat, a cross-field stimulation was applied to the posterior side of the ventricles, inducing reentry. Reentrant waves broke up due to the restitution properties of the myocardium, leading to VF. Simulations were run for 5 seconds post-VF induction to ensure that VF was sustained.

### Scroll-wave Filaments and Pseudo ECGs

To analyze the stability of the scroll waves, the number of scroll-wave filaments (the organizing centers of reentry) throughout the ventricular volume was determined at time instants 200 ms apart during 4 seconds of simulation using an algorithm based on phase angle maps, as described previously [37].

Pseudo ECGs were computed as follows [38]:
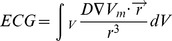
(2)where V is the ventricular volume, 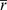 is the vector from each point in the ventricular volume to the recording electrode, the latter placed 10 cm from the center of the anterior wall of the ventricles in the anterior direction of the transverse plane, as done previously [14], and r is the distance from each point in the ventricular volume to the recording electrode.

## Results

### VF in the Electromechanical Model without SAC Representation

**Figure 2:**
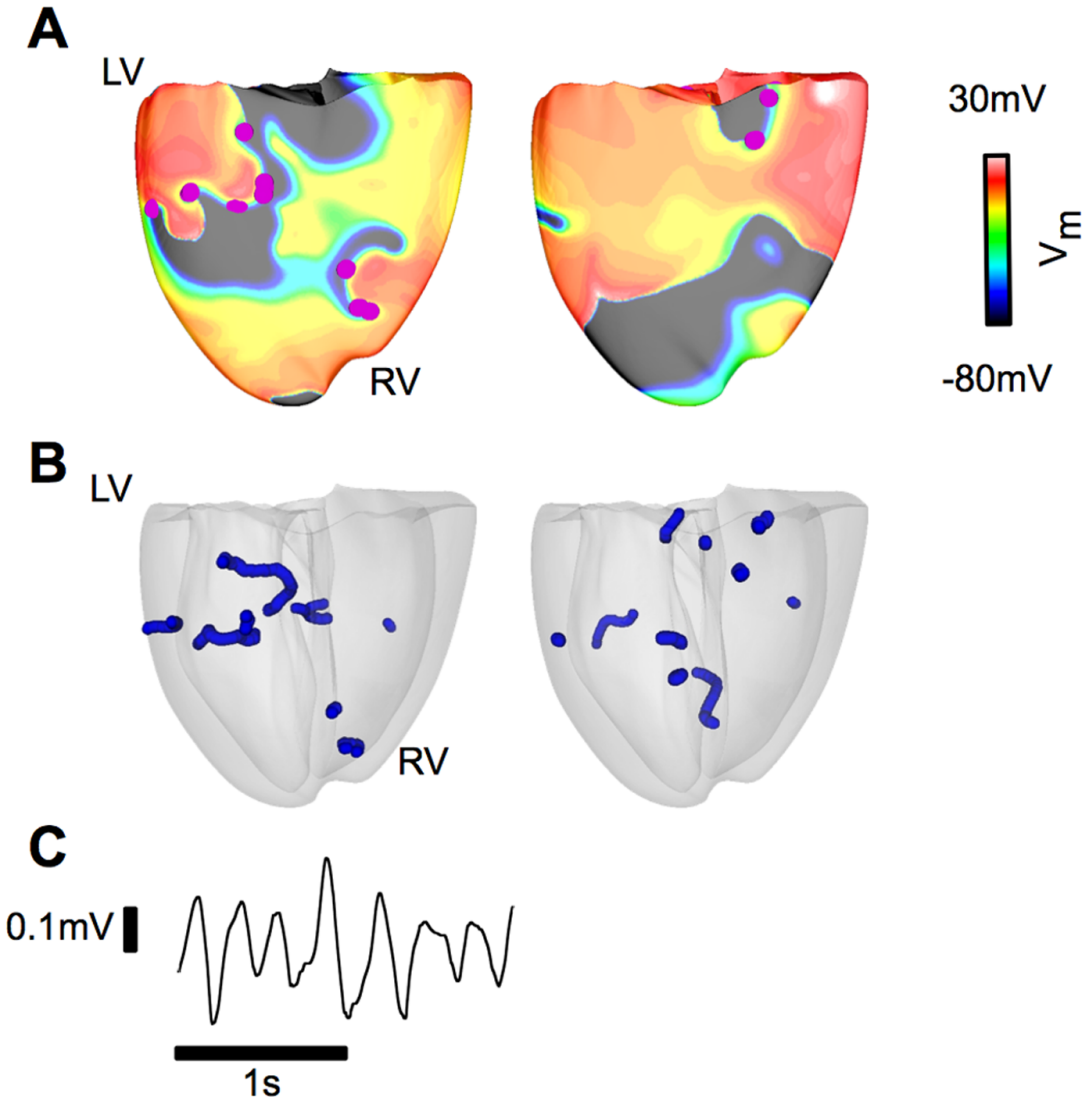
VF in the electromechanical model without SAC representation. (A): Epicardial transmembrane potential distribution maps on the posterior wall from the simulation without SAC representation. Pink dots indicate the locations of the phase singularities. (B): Posterior semi-transparent view of the ventricles shows the filament distribution (blue). (C): Pseudo-ECG.

Figure 2 presents the epicardial transmembrane potential distribution maps of sustained VF (Figure 2A) in the model without SAC representation; scroll waves break up continuously, maintaining VF (a Supplementary Movie (S1) is available in Supporting Information). There are multiple scroll wave filaments present in the ventricles during the simulation (Figure 2B). The irregular and complex pseudo-ECG (Figure 2C) is a manifestation of the numerous meandering reentrant waves sustaining VF.

### Inclusion of SAC with V_SAC_ of −60 mV Results in Partial Suppression of Scroll Wave Breakup by Flattening of the APD Restitution Curve

Comparing the number of filaments in the model with and without SAC (V_SAC_ of −60 mV**)** revealed that SAC activation partially suppressed (but did not eliminate) scroll wave breakup for all values of g_SAC_. Indeed, for the model with SAC, the average number of filaments decreased by 46–62%, depending on the value of g_SAC_, as compared to the model without SAC (Table 1A).

**Table 1:** The average number of filaments in the VF human ventricular electromechanical model with SAC of V_SAC_ of −60 mV for different g_SAC_.

	Average No. of filaments
Without SAC	7.6±2.2
g_SAC_ (mS/µF)	
0.03	2.9±1.0*
0.05	3.1±1.7*
0.07	4.1±1.7*

The symbol * indicates that the average number of filaments is significantly smaller than that in the model without SAC representation (p<0.05).

**Figure 3:**
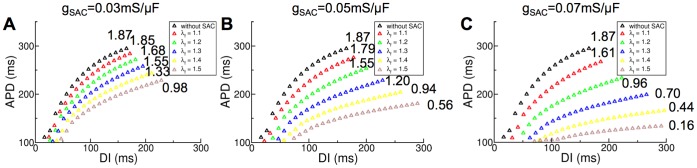
Recruitment of SAC with V_SAC_ of −60 mV flattens the single-cell APD restitution curve. Changes in the single-cell APD restitution curves due to SAC opening for different values of λ_f_. (A): g_SAC_ = 0.03 mS/µF, (B): g_SAC_ = 0.05 mS/µF and (C): g_SAC_ = 0.07 mS/µF.

**Figure 4:**
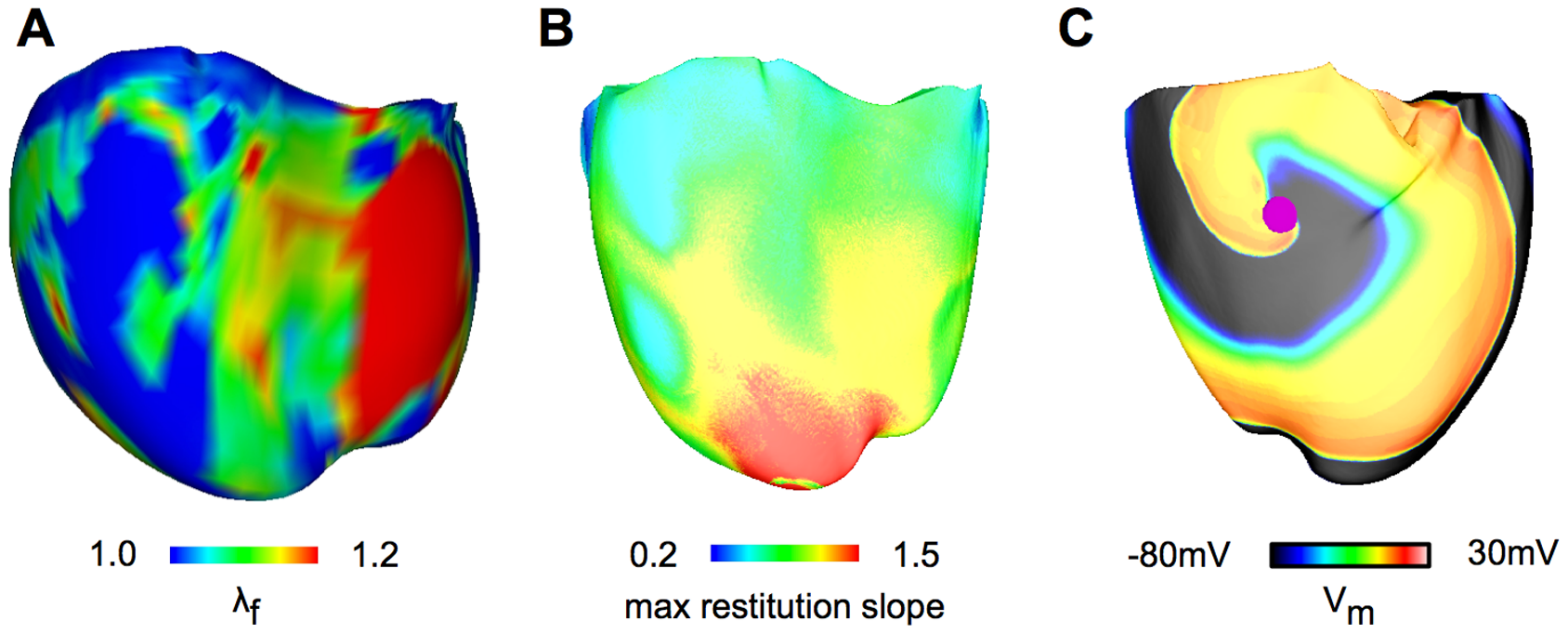
Recruitment of SAC with V_SAC_ of −60 mV diminishes scroll wave breakup. (A): Snapshot of the heterogeneous λ_f_ distribution at 2.3 s after arrhythmia induction for the ventricular model with V_SAC_ of −60 mV; g_SAC_ = 0.07 mS/µF. In plotting the λ_f_ distribution, the range 1.0 to 1.2 was chosen for visual purposes, as 90% of the data points fell within this range. (B): Maximum APD restitution slope distribution for the same model and time instant as in (A). In plotting of maximum APD restitution slopes, the range 0.2 to 1.5 was chosen for visual purposes, as 97% of the data points fell within this range. (C): Epicardial transmembrane potential distribution map on the anterior wall for V_SAC_ of −60 mV and g_SAC_ of 0.07 mS/µF when λ_f_ was assumed constant and equal to 1.2. Pink dot indicates the location of the phase singularity.

To understand the mechanisms by which recruitment of SAC with V_SAC_ of −60 mV decreased the likelihood of scroll wave breakup, we first investigated an important determinant of dynamic instability, the single cell APD restitution relation and its modification by MEF. Single cell APD restitution relations with SAC recruitment were calculated for the three values of g_SAC_ examined; results are presented in Figure 3. The strain map of the fibrillating human heart at each time instant during the simulation was analyzed (a representative strain map is shown in Figure 4A) and the maximum λ_f_ was found to be 1.5. Single-cell APD restitution curves were thus constructed for different degrees of SAC opening corresponding to λ_f_ values from 1.0 to 1.5. As shown in Figure 3, current through SAC leads to flattening of the single-cell APD restitution curve for all values of λ_f_ and g_SAC_. The larger the value of λ_f_ or g_SAC_, the flatter the resulting APD restitution curve.

At any time instant during VF, the distribution of λ_f_ in the ventricles was heterogeneous, as illustrated by the snapshot map in Figure 4A. This led to non-uniform I_SAC_ throughout the ventricles, which in turn gave rise to varying degrees of APD restitution flattening in the ventricular model with SAC. A map of the distribution of maximum restitution slope in the ventricles for g_SAC_ of 0.07 mS/µF is presented in Figure 4B. Regions of large λ_f_ had maximum restitution slopes smaller than 1, whereas regions of small λ_f_ had maximum restitution slopes larger than 1 but less than the original value of 1.8. These regional differences in the restitution-flattening effect of SAC opening are the reason why scroll waves continued to break up (albeit much less frequently). Should λ_f_ have been homogeneous and of value 1.2 or above, recruitment of SAC with V_SAC_ of −60 mV would have led to the conversion of VF into ventricular tachycardia, as our simulations found; Figure 4C shows a stable scroll-wave with a single filament throughout the simulation in this case (for λ_f = _1.2 everywhere).

### Recruitment of SAC with V_SAC_ of −10 mV Diminishes Scroll Wave Breakup at Low g_SAC_, but not at Large g_SAC_

Comparing the number of filaments that sustain VF in the ventricular model with and without SAC demonstrated that recruitment of SAC with V_SAC_ of −10 mV had a different effect on the stability of scroll waves depending on the value of g_SAC_. For low values of g_SAC_ (from 0.02 mS/µF to 0.04 mS/µF), the average number of filaments for the model with SAC decreased by 32–51% compared to that in the model without SAC (Table 2A), indicating less frequent scroll wave breakup. For large values of g_SAC_ (0.05 mS/µF and above), the average number of filaments for the model with SAC was not significantly different from that in the model without SAC (Table 2A), demonstrating that scroll wave breakup was not suppressed.

**Table 2:** The average number of filaments in the ventricular model with SAC of V_SAC_ of −10 mV for different g_SAC_.

	Average No. of filaments
Without SAC	7.6±2.2
g_SAC_ (mS/µF)	
0.01	7.3±3.1
0.02	4.4±1.6*
0.03	3.7±1.0*
0.04	5.2±2.1*
0.05	7.4±1.3
0.06	8.0±2.8
0.07	8.9±2.4

The symbol * indicates that the average number of filaments is significantly smaller than that in the model without SAC representation (p<0.05).

**Figure 5:**
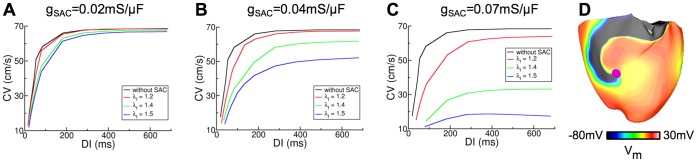
Recruitment of SAC with V_SAC_ of −10 mV diminishes scroll wave breakup at low g_SAC_. Changes in the CV restitution curves due to SAC opening for different values of λ_f_. (A): g_SAC_ = 0.02 mS/µF, (B): g_SAC_ = 0.04 mS/µF and (C): g_SAC_ = 0.07 mS/µF. (D): Epicardial transmembrane potential distribution map on the anterior wall for V_SAC_ of −10 mV and g_SAC_ = 0.04 mS/µF when λ_f_ was assumed constant and equal to 1.2. Pink dot indicates the location of the phase singularity.

To understand the mechanisms by which opening of SAC with V_SAC_ of −10 mV diminishes scroll wave breakup at low g_SAC_, we first determined the single cell APD restitution curves with SAC recruitment in this case and found that opening of SAC with V_SAC_ of −10 mV does not change the single cell APD restitution curves. Since previous studies have shown that flattening of conduction velocity (CV) restitution curves also leads to stabilization of scroll waves [39], [40], we next determined the CV restitution curves with SAC recruitment for all g_SAC_ values using a model of a slab of human ventricular tissue as done previously [41]. The results for three representative g_SAC_ values, 0.02, 0.04 and 0.07 mS/µF, are shown in Figure 5. Analysis of the ventricular strain maps for all time instants showed that in this case the maximum λ_f_ was also 1.5, despite the differences in the spatial distribution of strain. Thus, CV restitution curves were constructed for different degrees of SAC opening corresponding to λ_f_ of 1.0, 1.2, 1.4 and 1.5. As demonstrated in Figure 5A–C, current through SAC leads to flattening of the CV restitution curves for all values of λ_f_ and g_SAC_. The larger the value of λ_f_ or g_SAC_, the flatter the CV restitution curve.

Heterogeneous distribution of λ_f_ throughout the ventricles as demonstrated in Figure 4A gave rise to different degrees of SAC opening and thus CV restitution curve flattening. The flatter CV restitution curve in regions of substantial stretch resulted in suppression of scroll wave breakup there; the still-steep CV restitution curve in regions of minimal stretch continued to promote scroll wave breakup. As a result, the degree of spiral wave breakup and thus the number of filaments in the ventricles diminished. If the distribution of λ_f_ (of value 1.2 or above) were homogeneous, opening of SAC with V_SAC_ of −10 mV would have completely suppressed scroll wave breakup, as shown by the stable scroll waves throughout the simulation in Figure 5D (for λ_f = _1.2 everywhere).

**Figure 6:**
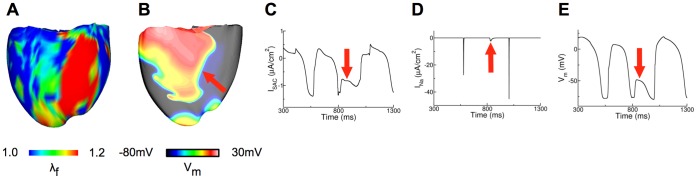
Recruitment of SAC with V_SAC_ of −10 mV results in scroll wave breakup at large g_SAC_. (A): Distribution of λ_f_ at 0.9 s after arrhythmia induction for the ventricular model with V_SAC_ of −10 mV; g_SAC_ = 0.07 mS/µF. (B): Scroll wave breakup in the region of large stretch (indicated by arrow). (C), (D) and (E) are plots of I_SAC_, I_Na_ and V_m,_ respectively from the node indicated by the arrow in (B). The arrow denotes in (C): the large inward I_SAC_ during repolarization, in (D): inactivation of Na channels, (E): conduction block.

For large values of g_SAC_, opening of SAC with V_SAC_ of −10 mV in regions of substantial stretch (Figure 6A) resulted in a large inward I_SAC_ during repolarization (Figure 6C), which elevated the resting membrane potential from −85 mV in the model without SAC to −77 mV in the ventricles with SAC (Figure 6E) and thus inactivated the Na channels in the latter model (Figure 6D). As a result, conduction block occurred in regions of substantial stretch (Figure 6E) causing scroll wave breakup there (Figure 6B). The scroll wave breakup in regions of substantial stretch counteracted the increased scroll wave stability due to a flatter CV restitution there (Figure 5C), explaining why the number of scroll wave filaments in the model with SAC was not significantly different from that in the model without SAC at large values of g_SAC_.

## Discussion

This study investigated the effects of SAC opening on scroll wave stability in the fibrillating ventricles by employing a strongly-coupled MRI-based anatomically accurate 3D model of human ventricular electromechanics. A comprehensive analysis of how recruitment of SAC influences scroll wave breakup was performed for different SAC reversal potentials and channel conductances. We discovered that recruitment of SAC affects scroll wave stability via different mechanisms depending on the reversal potential and channel conductance of SAC.

1. Opening of SAC with V_SAC_ of −60 mV decreases the likelihood of scroll wave breakup for all values of g_SAC._ The underlying mechanism is flattening of the APD restitution curve in regions of high strain.
2. Opening of SAC with V_SAC_ of −10 mV partially inhibits scroll wave breakup at low values of g_SAC_ by flattening the CV restitution relation in regions of high stretch. For large values of g_SAC_, recruitment of SAC with V_SAC_ of −10 mV did not diminish the likelihood of scroll wave breakup because Na channel inactivation in regions of large stretch (as a result of SAC opening) led to conduction block and thus scroll wave breakup, which counteracted the increased scroll wave stability due to a flatter CV restitution.

### The MRI-based Electromechanical Model of the Human Ventricles

In this study, we present a strongly-coupled MRI-based model of human cardiac electromechanics. This new model incorporates reconstructions of human ventricular geometry and fiber orientation from MR and diffusion tensor MR images, which allows for simulation of realistic ventricular deformation during arrhythmias. The circulatory model to which it is coupled is adapted for the human ventricles, enabling accurate representation of hemodynamic changes during arrhythmia. The implementation of strong coupling in our electromechanical model allows for the dynamic inclusion of the effect of MEF in the electrical component since a mechanical solution step was performed following every fifth electrical solution step; this allows for a more accurate simulation of the effect of MEF compared to what has been done previously, where a mechanical solution step followed every 100 electrical solution steps [14]. This is the first electromechanical model to incorporate MEF in this manner. The simulations performed using this model in the present study represent a comprehensive evaluation of the electromechanical behavior of the human ventricles in VF and of the effect of SAC opening on arrhythmia stability.

### Alteration of Cellular Electrophysiological Properties by MEF

Recruitment of non-selective cation SAC results in prolongation of APD with a crossover during systole, where the early phase of repolarization is shortened and the late phase prolonged [10], [32]. Opening of potassium-conducting SAC causes repolarization during systole and results in APD shortening [16]. Thus, the combined electrophysiological effect of the opening of the two different SACs can vary depending on the degree of expression of non-selective cation SAC and potassium-conducting SAC in myocardial tissue; experimental observations have shown both prolongation [17], [32], [42] and shortening of APD or monophasic action potential [43], [44].

Single cell behavior in our models is consistent with this experimental data, as SAC opening with V_SAC_ at −60 mV, which represented a higher degree of expression of potassium-conducting SAC, produced shortening of APD. SAC opening with V_SAC_ at −10 mV, which represented a higher degree of expression of non-selective cation SAC, resulted in APD prolongation with a crossover and elevation of resting potential. Different conductances have been reported for SAC as well [5]; we showed that the degree of lengthening or shortening of APD is affected by SAC conductance. Since recruitment of SAC with different reversal potentials and conductances leads to different electrophysiological changes in cardiac myocytes, it was important to incorporate different degrees of expression of the two types of SAC when examining the whole-heart behavior, extending the findings of an earlier study [14].

### Effects of MEF on CV

While examining the dependence of CV on strain was not the subject of this study, this relationship affected the dependence of the CV restitution on MEF, and thus indirectly spiral wave behavior in the model. Previous studies have shown that for a wide range of pacing cycle lengths, the CV can exhibit biphasic; constant; increasing; and decreasing relationship with respect to strain (see review [11]). Our results are consistent with these seemingly disparate findings: we found that CV displays different relationships with respect to stretch depending on pacing cycle length, g_SAC_, and V_SAC_. Opening of SAC with V_SAC_ of −10 mV resulted in independence of CV on stretch for low values of g_SAC_ and in a decrease in CV with the increase in stretch for large values of g_SAC_ at all pacing cycle lengths. For low values of g_SAC_, opening of SAC with V_SAC_ of −10 mV produced a small inward I_SAC_ during repolarization, which did not lead to large elevation in the resting membrane potential and thus did not inactivate Na channels and reduce CV. For large values of g_SAC_, recruitment of SAC with V_SAC_ of −10 mV resulted in a larger I_SAC_, which was sufficient to inactivate Na channels and thus slow conduction. Recruitment of SAC with V_SAC_ of −60 mV resulted in the increase in CV with increasing stretch at short pacing cycle lengths (400 ms and below) and in a CV unaltered with stretch at long pacing cycle lengths (between 400 and 1000 ms) for all values of g_SAC_. Opening of SAC with V_SAC_ of −60 mV shortened APD and thus increased DI. At short pacing cycle lengths, the increase in DI led to a better recovery from refractoriness and thus increased CV, whereas at large pacing cycle lengths, there was already a full recovery from refractoriness without SAC opening and thus the increase in DI did not increase CV.

### Effect of SAC Recruitment on Scroll Wave Stability

There has been a significant body of research on the determinants of scroll wave stability. APD and CV restitution relationships have been found to be two main determinants of scroll wave stability; a flat APD or CV restitution curve was shown to lead to stable scroll waves, whereas a steep APD or CV restitution relation gives rise to scroll wave breakup [39], [45], [46]. Studies concerning determinants of scroll wave stability have mainly been electrophysiological without taking into account the effect of mechanical contraction of the ventricles on scroll wave stability. However, heterogeneity in strain throughout the ventricles, especially during VF, leads to heterogeneous MEF via SAC opening, which affects scroll wave stability. Since experimental studies require contraction to be blocked to reduce movement artifacts from optical mapping recording [47], realistic modeling offers a means to explore how mechanical contraction of the ventricles affects scroll wave stability via MEF.

We showed that opening of SAC with V_SAC_ of −60 mV diminished scroll wave breakup by flattening the APD restitution curve. Our findings are consistent with experimental results on scroll wave stability in the presence of the drug D600 (a calcium channel blocker at low concentrations), which caused acceleration of repolarization and shortening of APD, effects similar to those of SAC opening with V_SAC_ of −60 mV: D600 similarly promoted scroll wave stability by flattening the APD restitution curve [13].

We also demonstrated that opening of SAC with V_SAC_ of −10 mV partially inhibits scroll wave breakup at low values of g_SAC_ by flattening the CV restitution relation in regions of high stretch. Previous studies have shown that slowing of scroll wave rotation leads to stabilization of scroll waves [23], [48], [49]; the mechanism is that the period of rotation and thus the diastolic interval increase, resulting in the operational regime being in the less steep part of the APD restitution curve [48], [49]. This indirect suppression of scroll wave breakup is also present in our simulation results, as Na channels were inactivated with opening of SAC with V_SAC_ of −10 mV and thus scroll wave rotation was slowed.

For large values of g_SAC_, recruitment of SAC with V_SAC_ of −10 mV did not diminish the likelihood of scroll wave breakup because Na channel inactivation in regions of large stretch as a result of SAC opening led to conduction block and thus scroll wave breakup, which counteracted the increased scroll wave stability due to a flatter CV restitution. This is consistent with results obtained by Keldermann et al.’s [14]. Since Keldermann et al. evaluated the effect of large SAC conductances only, the destabilizing effect of SAC opening on scroll waves was the dominant mechanism. The study by Kuijpers et al. [50] demonstrated that in the atria, Na channel inactivation as a result of SAC opening leads to functional block, thereby terminating arrhythmias. The fact that Na channel inactivation as a result of SAC opening led to scroll wave breakup in the ventricles, whereas in atria it led to termination of arrhythmias might be due to the fact that atria have less tissue for propagation compared to the ventricles. Atria, unlike the ventricles, may not be able to support an alternative pathway circumventing the conduction block that is long enough, compared to the wavelength, to result in the establishment of a sustained reentry.

The results of the study demonstrate the possible therapeutic potential of SAC recruitment during VF, indicating that clinical strategies could be devised to minimize scroll wave breakup. For instance, gene therapy could be designed and tested to increase the expression of potassium-conducting SACs, so that g_SAC_ would be increased while V_SAC_ is brought closer to the myocyte resting potential. This will bring SACs to a regime that maximizes the suppression of scroll wave breakup.

### Study Limitations

Previous experiments have shown that SAC conductance can be a function of strain rate [51], [52]. However, this limitation would not greatly affect our results since it was previously shown that regions with larger strains were associated with larger strain rates [53]. SAC was assumed to be uniformly distributed in the ventricles. Such assumption was made due to lack of experimental studies on this subject.

## Supporting Information

Table S1 **Adjusted parameters of the Kerckhoffs et al. circulatory model.**
(DOCX)

Movie S1 **VF in the ventricular model without SAC representation.**
(MP4)
