# Supplementary material for: Effects of Mechano-Electric Feedback on Scroll Wave Stability in Human Ventricular Fibrillation
Source: PLoS One. 2013 Apr 3;8(4):e60287. doi: 10.1371/journal.pone.0060287 (PMC3616032; doi:10.1371/journal.pone.0060287)
Supplement: Table S1 — Adjusted parameters of the Kerckhoffs et al. circulatory model. (DOCX) [file pone.0060287.s001.docx]

| Time-varying elastance atrial model | |
| --- | --- |
| E_LA,max_ | 0.0782 kPa/ml |
| E_LA,min_ | 0.0711 kPa/ml |
| V_LA,rd_ | 64.6 ml |
| V_LA,rs_ | 60 ml |
| E_RA,max_ | 0.03 kPa/ml |
| E_RA,min_ | 0.0273 kPa/ml |
| V_RA,rd_ | 64.6 ml |
| V_RA,rs_ | 60 ml |
| Systemic circulation | |
| R_mitral_ | 1*10^-4^ kPa*s/ml |
| R_ao_ | 0.007 kPa*s/ml |
| R_as_ | 0.124 kPa*s/ml |
| R_vs_ | 0.02 kPa*s/ml |
| C_as_ | 97.8 ml/kPa |
| C_vs_ | 1299 ml/kPa |
| Pulmonic circulation | |
| R_tricus_ | 1*10^-4^ kPa*s/ml |
| R_pa_ | 0.004 kPa*s/ml |
| R_ap_ | 0.011 kPa*s/ml |
| R_vp_ | 0.011 kPa*s/ml |
| C_ap_ | 125.1 ml/kPa |
| C_vp_ | 150.0 ml/kPa |
